# Validation of brownie baking step for controlling *Salmonella* and *Listeria monocytogenes*

**DOI:** 10.1002/fsn3.2132

**Published:** 2021-01-21

**Authors:** Phoebe Unger, Lakshmikantha H. Channaiah, Arshdeep Singh, Amninder Singh Sekhon, Monipel Babb, Yaeseol Yang, Minto Michael

**Affiliations:** ^1^ School of Food Science Washington State University Pullman WA USA; ^2^ AIB International Manhattan KS USA

**Keywords:** baking, brownies, *D*‐values, thermal inactivation

## Abstract

Pathogens, such as *Salmonella* and *Listeria monocytogenes*, can survive under the dry environment of flour for extended periods of time and could multiply when flour is hydrated to prepare batter or dough. Therefore, inactivation of these pathogens during the cooking/baking step is vital to ensure the microbiological safety of bakery products such as brownies. The aim of this research was to validate a simulated commercial baking process as a kill‐step for controlling *Salmonella* and *L. monocytogenes* in brownies and to determine thermal inactivation parameters of these pathogens in brownie batter. Independent studies were conducted in a completely randomized design for each pathogen. All‐purpose flour was inoculated with a 5‐serovar *Salmonella* and 3‐strain *L. monocytogenes* cocktails. For baking validation, brownie batters were prepared from inoculated flour, and cooked in the oven set at 350°*F* (176.7°C) for 40 min followed by 15 min of ambient air cooling. For calculating *D*‐values, brownie batter was transferred into thermal‐death‐time disks, sealed, and placed in hot‐water baths. The samples were held for pre‐determined time intervals in hot‐water baths and immediately transferred to cold‐water baths. Microbial populations were enumerated using injury‐recovery media. At the end of baking, *Salmonella* and *L. monocytogenes* populations decreased by 6.3 and 5.9 log CFU/g, respectively. *D*‐values of *Salmonella* and *L. monocytogenes* cocktails were 53.4 and 37.5 min at 64°C; 27.2 and 16.9 min at 68°C; 10.7 and 9.1 min at 72°C; and 4.6 and 7.3 min at 76°C; respectively. The *z*‐values of *Salmonella* and *L. monocytogenes* cocktails were 11.1 and 16.4°C, respectively. This study can be used as a supporting document for the validation of similar brownie baking processes to control *Salmonella* and *L. monocytogenes*. The data from this study can also be employed for developing basic prediction models for the survival and thermal resistance of these pathogens during brownie baking step.

## Graphical abstract

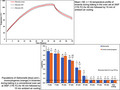

## 1 INTRODUCTION

The U.S. Food and Drug Administration's Food Safety Modernization Act (FSMA) is an important step taken toward making the food supply chain in the United States safer by striving to prevent foodborne illnesses rather than responding to the foodborne illness outbreaks (FDA, 2018a). The FSMA requires that food processor should have science‐based food safety as well as food defense plans to prevent accidental and intentional contamination (FDA, 2018a; Kennedy et al., 2014). According to the FSMA, the preventive control processing steps should be validated on the basis of scientific studies to ensure that identified hazards are controlled or eliminated (FDA, 2018b).

Although thermal processing steps during food preparation are considered effective to control or eradicate foodborne pathogens, these thermal kill‐step steps need to be validated to ensure that every food particle is exposed to the minimum required temperature for the minimum required time to effectively control the identified microbiological hazard. Contamination in foods can be introduced from various sources including raw ingredients, processing environment, employees, and/or post‐processing steps. Foodborne pathogens, such as *Salmonella* and *Listeria monocytogenes*, cannot grow in low water activity (*a*_w_) food ingredients (such as flour, powders, and spices), but they can survive for months to years in dry environments (Ballom et al., 2020; Beuchat et al., 2013; Taylor et al., 2018). Pathogens dormant in dry ingredients could start multiplying when rehydrated to prepare batter or dough, and could cause illnesses if appropriate preventive control steps are not effective. Therefore, it is vital for the food industry to validate all implemented preventive controls to ensure the safety of the finished food products.

The main objective of this research was to validate a simulated commercial brownie baking process to control *Salmonella* and *L. monocytogenes* in brownies using a conventional kitchen oven. Dry ingredients such as all‐purpose flour used in the production of brownies could introduce microbial contamination to the brownie batter as flour does not have any post‐process lethality treatment (Myoda et al., 2019). *Salmonella* and *L. monocytogenes* were used in this validation study because they are two of the most common non‐spore former foodborne pathogens that can survive in dry environments for prolonged periods of time. Moreover, these pathogens are also two of the top five pathogens responsible for foodborne‐related illnesses and/or deaths in the United States (Scallan et al., 2011). *Listeria monocytogenes* represented Gram positive bacterial group, whereas *Salmonella* represented Gram negative group.

## 2 MATERIALS AND METHODS

### 2.1 Experimental and statistical design

This research consisted of two independent studies: (a) brownie baking process validation: consisted of two individual validation experiments using artificially inoculated flour to control *Salmonella* and *L. monocytogenes* in brownies during baking, and product water activity (*a*_w_) and pH were monitored during the baking as well; (b) *D*‐ and *z*‐values determination: consisted of two individual experiments to determine thermal inactivation parameters of *Salmonella* and *L. monocytogenes* in brownie batter prepared from artificially inoculated flour.

For the validation studies, brownie batter was prepared from inoculated flour, baked, aseptically sampled, and enumerated for the respective pathogen populations. These studies were conducted as completely randomized design with ten treatments (pre‐baking; 5, 10, 15, 20, 25, 30, 35, and 40 min sampling times during baking, and 15 min of ambient cooling post‐baking) and three replications. One‐way ANOVA using Minitab® 19 was used to determine any statistical differences (α = 0.05) in the microbial populations, a_w_ and pH. The *D*‐ and *z*‐values studies were also designed as completely randomized with three replications, and *D*‐values of *Salmonella* and *L. monocytogenes* in brownie batter were determined at 64, 68, 72, and 76°C. The linear regression graphs were plotted using Microsoft® Excel 2018 (Version 16.20; Microsoft® Corporation), and statistical differences in *D*‐ and *z*‐values were determined using one‐way ANOVA using Minitab® 19.

**TABLE 1:** Bacterial cultures used in the study

Pathogen	ATCC® No.	Isolated from	ATCC® Recommendation	Serovar
*Salmonella* serovars	43,845	N/A	Enteric research	Senftenberg
	14,028	Chicken	Enteric research, water & media testing, control culture	Typhimurium
	BAA‐708	Human associated with egg‐borne illness	Enteric research, disinfectant testing	Enteritidis
	6962	Food poisoning fatality, England	Enteric research	New port
	BAA‐710	Human associated with tomato‐borne illness	Enteric research, disinfectant testing	Montevideo
*Listeria monocytogenes*	5,414	Raw milk outbreak in Massachusetts	Enteric research	‐
	19,115	Human	Enteric research, quality control	‐
	19,111	Poultry, England	Enteric research, food & media testing	‐

ATCC®: American Type Culture Collection (Manassas, VA).

### 2.2 Microbial cultures

All microbial cultures were obtained from the American Type Culture Collection (ATCC®; Manassas, VA) in freeze‐dried forms. Five serovars of *Salmonella* and three strains of *L*. monocytogenes were used in the current research (Table 1). These cultures were selected based on their isolation from various food matrices and/or association with foodborne illnesses. All cultures were propagated according to the manufacturer's instructions, and working cultures were stored in 10 ml of Brain Heart Infusion (BHI; Difco™, Becton, Dickinson and Company) broth at 4°C.

### 2.3 Master inoculation preparation

For each replication, individual serovars/strains of *Salmonella* and *L. monocytogenes* were streaked from BHI broth onto BHI agar as lawns using sterile swabs and incubated at 37°C for 24 hr. Lawns of individual strains were harvested using 1 ml of 0.1% peptone solution (Bacto™, Becton, Dickinson and Company) twice and dislodging bacterial cells using sterile plastic L‐spreaders (Michael et al., 2020). The harvested individual bacterial solutions were then mixed in equal proportion to obtain a 5‐serovar cocktail of *Salmonella* or 3‐strain cocktail of *L. monocytogenes*, transferred into 50 ml sterile plastic tubes connected to spray nozzles, and used as master inoculum for inoculating the flour. The spray nozzles were calibrated to discharge ~1 ml of master inoculum per squirt. The lawn method was used for the master inoculum preparation because it produced highly concentrated bacterial cell solution, which resulted in less volume of inoculum required for mist inoculating the flour.

### 2.4 Flour inoculation

Two hundred grams of all‐purpose wheat flour was weighed in a sanitized sealable plastic tub (~38 × 26 × 7 cm, Rubbermaid Inc.) and spread evenly to get a uniform layer. The flour in tub was then transferred inside a biosafety cabinet and mist inoculated by spraying ~4 ml of master inoculum in four squirts. Inoculated flour was then dried back to the original pre‐inoculation weight by incubating with open lids at 37°C for ~4 hr to achieve the a_w_ of 0.305 ± 0.01. The dried, inoculated flour was mixed well using a sanitized spatula, sealed with air‐tight lids, and stored at room temperature (~20°C) until used within 2 days.

### 2.5 Brownie batter preparation

All of the ingredients were purchased from a local store in Pullman, WA. The Brownie recipe including all the baking parameters used in the study was based on a general recipe used for commercial bakery brownies. Inoculated flour was weighed in a sanitized mixing bowl followed by the addition of granulated sugar, powdered sugar, cocoa powder, salt, soybean oil, water, fresh liquid whole egg, and vanilla extract (Table 2). The mixing bowl with ingredients was then attached to a kitchen mixer (Classic KitcehnAid®) and mixed for 1 min at speed‐2 with scrapping after first 30 s.

**TABLE 2:** Brownie recipe used in the study

Ingredient	Grams
Flour	300
Sugar	610
Cocoa powder	160
Salt	2
Powdered sugar	70
Water	30
Soybean oil	200
Whole egg	300
Vanilla extract	20

### 2.6 Brownie baking

**FIGURE 1:**
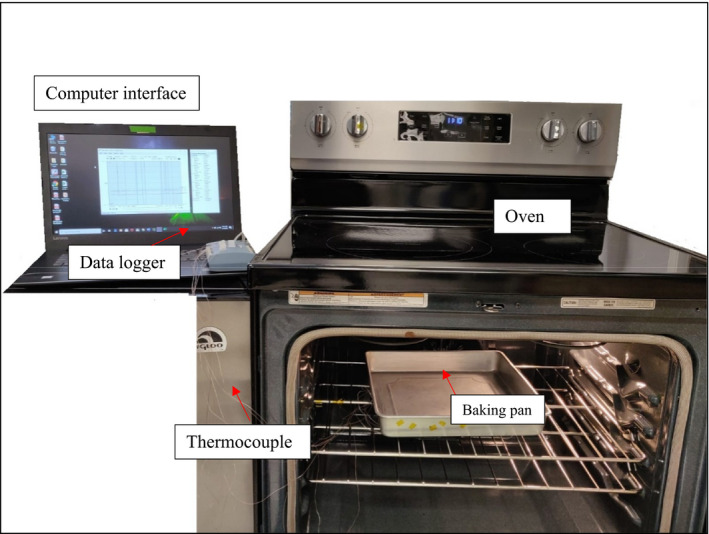
Set up used for brownie baking validation study

**FIGURE 2:**
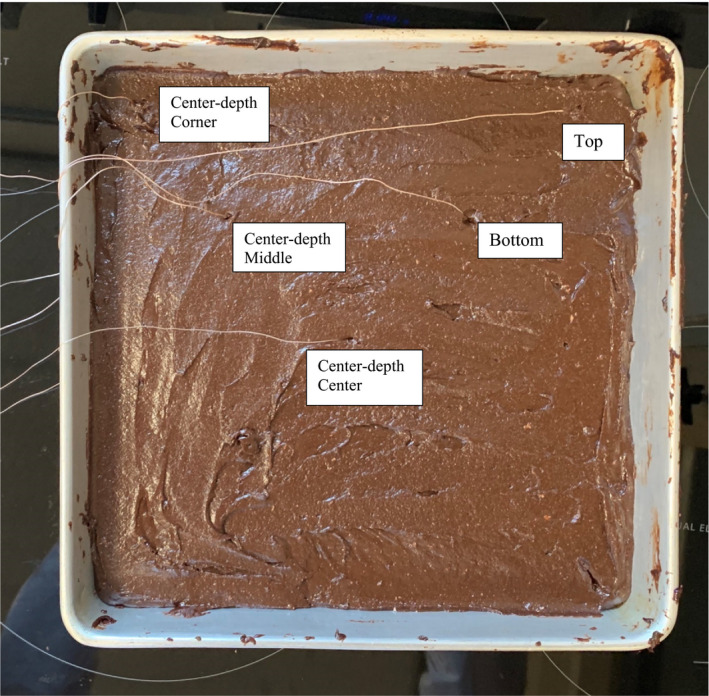
Uncooked brownie batter connected with five thermocouples placed in various locations

Brownie batter (~1,690 g) was evenly spread [~1.25 cm height] using a sanitized spatula into a 12 × 12 inch (30.48 × 30.48 cm) pan and placed into a conventional oven preheat to 350°F (176.7°C) (Figure 1). Five T40 fine‐gauge type‐K thermocouples (Thermo‐Electra) were placed in the brownie batter, and one was placed inside the oven. The thermocouples in brownie batter were placed in 5 different spots throughout the pan: at the geometric center, at the center depth halfway from the edge of the pan to the center, at the center depth in the corner, at the top surface of brownies, and at the bottom of the brownies (Figure 2). All six thermocouples were connected to a data logger (USB‐TC Measurement Computing™) and a computer. The timer was started as soon brownie batter was placed in the oven, and the brownies were sampled every 5 min. After 40 min of baking, baked brownies were removed from the oven, followed by 15 min of ambient air cooling. During the preliminary work, it was confirmed that opening the oven door for <10 s for sampling did not affect the product temperature. However, although the oven air temperature did decrease during the sampling process, this decreased oven air temperatures reached back to the pre‐sampling temperatures in <45 s.

### 2.7 *a*_w_ and pH determination

The *a*_w_ of samples was measured at 25°C using a calibrated a_w_ meter (Novasina Labswift Portable *a*_w_ meter). The *a*_w_ was measured for the batter prepared from inoculated flour at 0, 5, 10, 15, 20, 25, 30, 35, 40, and after a 15 min ambient air cooling. For measuring a_w_ during baking, one sample was taken at each sampling time. Until 20 min, *a*_w_ was measured for the whole brownie batter sample. However, from 20 min, there was a noticeable difference in appearance of the crust and crumb; therefore, the crumb and crust were separated and used to measure *a*_w_ separately. Samples were aseptically transferred into *a*_w_ cups, sealed, and allowed to cool to ~25°C before measuring *a*_w_. After measuring *a*_w_, sample pH was also measured using a calibrated surface pH meter (ExStik® Waterproof pH Meter, Extech) at 25°C.

### 2.8 Brownie baking validation

For the brownie baking validation study, microbial enumeration of the respective pathogen was conducted for inoculated flour, batter, 5 min intervals during baking, and after 15 min of ambient air cooling. At each sampling point, ~15 g samples were transferred into stomacher bags (~13.5 × 20.5 cm, Nasco Whirl‐Pak®) containing chilled (~4°C) 45 ml of 0.1% peptone solution, stomached for 1 min (using Smasher™, Ace Laboratore), and stored at 4°C until analyzed within 30 min.

Although the actual baking time to achieve best quality brownies was baking for 40 min, samples were taken at 5 min intervals during baking to account for worst‐case scenarios where brownies might be undercooked because of cooking process malfunctioning. As microbial kill in brownies can continue during ambient cooling, a 15‐min ambient cooling treatment was also included in the studies.

### 2.9 D‐ and z‐values determination

**FIGURE 3:**
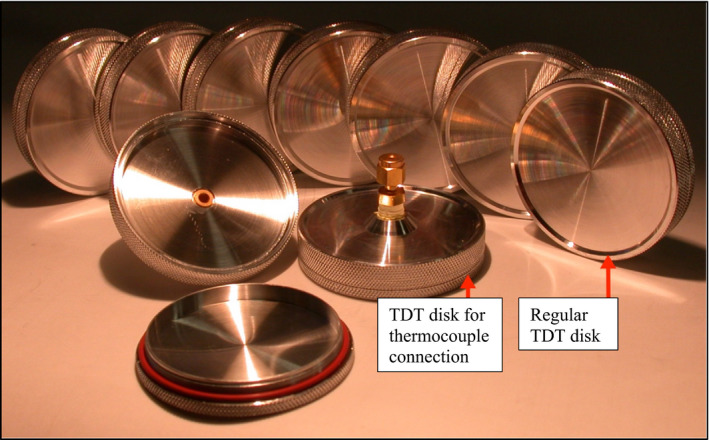
Aluminum thermal‐death‐time (TDT) disks (internal height of 5 mm and diameter of 50 mm) used for determining *D*‐values of *Salmonella* and *L. monocytogenes* in brownie batter

*D*‐values were determined at 64, 68, 72, and 76°C following the method described by Channaiah et al. (2018) using temperature‐controlled water baths (18 L, Grant Instruments Ltd.) and sanitized aluminum thermal‐death‐time (TDT) disks (Engineering Shop, Washington State University). The TDT disks had internal height and diameter of 5 and 50 mm, respectively (Figure 3). Batter prepared from inoculated flour was transferred into four regular and one T‐type thermocouple‐connected TDT disks (10 ± 0.1 g in each TDT disk). The five TDT disks containing brownie batter were placed inside a small plastic crate (15.6 × 20 × 22.9 cm; Sterilite®) and then transferred into hot‐water baths pre‐set at the respective temperatures. Once the batter inside the TDT disks achieved the target temperature, the first TDT disk was removed from the water bath and placed in ice‐water bath (~1°C) and quickly cooled to ~4°C. The other four TDT disks were then randomly removed at the pre‐determined sampling times, with TDT disk connected to the thermocouple removed at the end. The temperature of brownie batter inside TDT disks was monitored using a data logger (Fluke 51‐2 Thermometer). At each sampling point, samples were transferred into stomacher bags (~6.5 × 10.5 cm, Nasco Whirl‐Pak®) containing 10 ml of 0.1% peptone solution, stomached for 1 min (using Seward Stomacher® 80 Lab System), and stored at 4°C until analyzed within 30 min. Thermal inactivation graphs of individual pathogens at respective temperatures were plotted between log CFU/g population and corresponding time (min), and *D*‐values were calculated as the absolute values of the inverse of the slopes. For *z*‐values calculations, linear regression graphs for the individual pathogens were plotted between the log *D*‐values and corresponding temperatures, and *z*‐values were calculated as the absolute values of the inverse of the slopes.

### 2.10 Microbial enumeration

Microbial populations of each pathogen during the validation and *D*‐values studies were enumerated on the respective injury‐recovery media. Samples in stomacher bags were serially diluted using 0.1% peptone solution and spread plated on BHI agar. The BHI agar plates were incubated for ~4 hr at 37°C, overlaid with the selective media, and then further incubated for 20 hr for *Salmonella*, and 36 hr for *Listeria* at 37°C. The selective plating medium used for *L. monocytogenes* was PALCAM (polymyxin B, acriflavine, lithium chloride, ceftazidime, aesculin, D‐mannitol) agar supplemented with PALCAM antimicrobic supplement (Difco™) (Hitchens et al., 2017). Whereas, XLD (xylose lysine deoxycholate; Difco™) agar was used as the selective medium for the *Salmonella* enumeration (Andrews et al., 2019).

## 3 RESULTS AND DISCUSSIONS

### 3.1 Brownie baking validation

**FIGURE 4:**
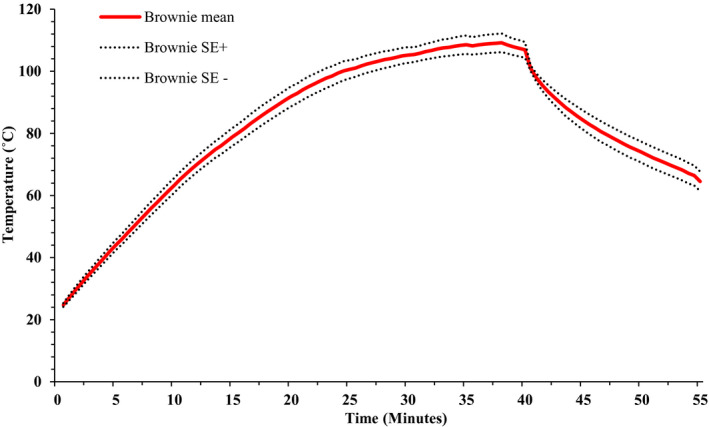
Mean (± *SE*; *n* = 6) temperature profile of brownie during baking in the oven set at 350°F (176.7°C) for 40 min followed by 15 min of ambient air cooling

**FIGURE 5:**
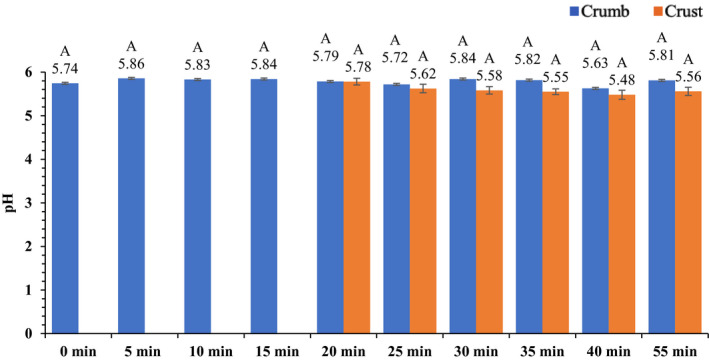
Mean (± *SE*) pH of brownies during 40 min of baking and after 15 min of ambient air cooling

**FIGURE 6:**
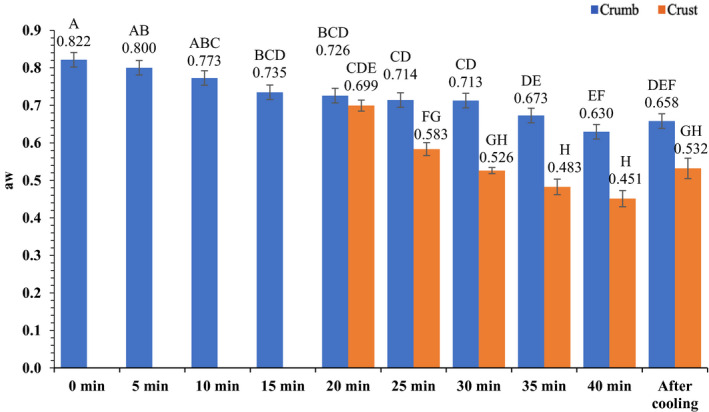
Mean (± *SE*) water activity (*a*_w_) of brownies during 40 min of baking, and after 15 min of ambient air cooling

The average temperature of the brownies during baking is presented in Figure 4. After 10 min of heating, the average brownie temperature increased from 22.2 ± 0.31 to 63.3 ± 2.39°C. After 20 min of baking, the brownie temperature had increased to 91.9 ± 3.23°C. After 40 min of baking, the complete bake time, the temperature was 106.9 ± 2.54°C. At the end of 15 min of ambient air cooling, the brownie temperature decreased to 64.5 ± 3.12°C. The average pH and a_w_ throughout the baking process are presented in Figures 5 and 6, respectively. Overall, pH of brownies did not change during the baking process. The pH of brownie batter at the start of baking was 5.7 ± 0.078; whereas, at the end of ambient cooling, brownie pH was 5.6 ± 0.094 and 5.8 ± 0.060 in crust and crumb, respectively. The *a*_w_ of brownie batter at the start of baking was 0.822 ± 0.007, which significantly decreased after ambient air cooling, with *a*_w_ of 0.658 ± 0.013 in brownie crumb and 0.532 ± 0.027 in brownie crust.

**FIGURE 7:**
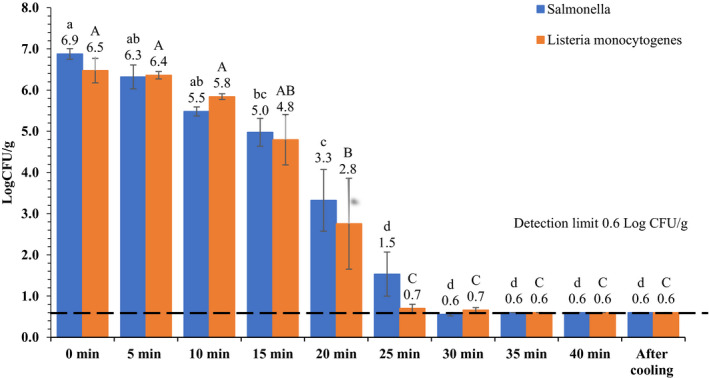
Populations of *Salmonella* and *L. monocytogenes* in brownies during baking in a conventional oven set at 350°F (176.7°C) for 40 min followed by 15 min ambient air cooling

Populations of *Salmonella* and *L. monocytogenes* in brownies during the baking and after ambient air cooling are presented in Figure 7. *Salmonella* populations decreased significantly at 15, 20, 25, 30, 35, 40 min and after cooling compared to the original population in the batter. *L. monocytogenes* populations decreased significantly at 20, 25, 30, 35, 40 min and after cooling compared to the original population in the batter. *Salmonella* and *L. monocytogenes* levels in the inoculated flour were 7.4 ± 0.12 and 7.3 ± 0.25 log CFU/g, respectively. Whereas, *Salmonella* and *L. monocytogenes* levels in the brownie batters were 6.9 ± 0.13 and 6.5 ± 0.30 log CFU/g, respectively. Baking brownies for 40 min resulted in ≥ 6.3 ± 0.13 and ≥ 5.9 ± 0.30 log reductions in *Salmonella* and *L. monocytogenes* populations, respectively, with microbial populations decreased below the enumeration detection limit (0.6 log CFU/g). As pathogenic contamination in food products in real life scenarios could rarely be >2 log CFU/g, baking brownie at 176.7°C oven temperature for ≥25 min would result in >5‐log reductions in *Salmonella* and *L. monocytogenes* populations, ensuring microbial safety of brownies.

Channaiah et al. (2016) conducted a similar validation study to control *Salmonella* (Typhimurium, Newport and Senftenberg 775W) in simulated commercial hamburger bun baking process at 218.3°C oven temperature. They reported that by the end of 13 min of baking (~100°C internal bun temperature), >6 log reductions were achieved for all three *Salmonella* serovars. In the same study, Channaiah et al. (2016) also studied the inactivation of *Enterococcus faecium* during the baking process. Channaiah et al. (2016) concluded that as *E. faecium* took longer to achieve >6 log reduction and exhibited greater thermal resistance compared to *Salmonella*, *E. faecium* can be used as a surrogate for *Salmonella* for in‐plant validation studies. In another validation study, Channaiah et al. (2018) reported that frying donuts in oil at 190.6°C for 2 min (1 min on each side) resulted in >7 log reductions in 7‐serovar *Salmonella* cocktail population (Newport, Typhimurium, Senftenberg, Tennessee and three dry food isolates) and the internal donut temperature reached to ~119°C.

### 3.2 D‐ and z‐values

**FIGURE 8:**
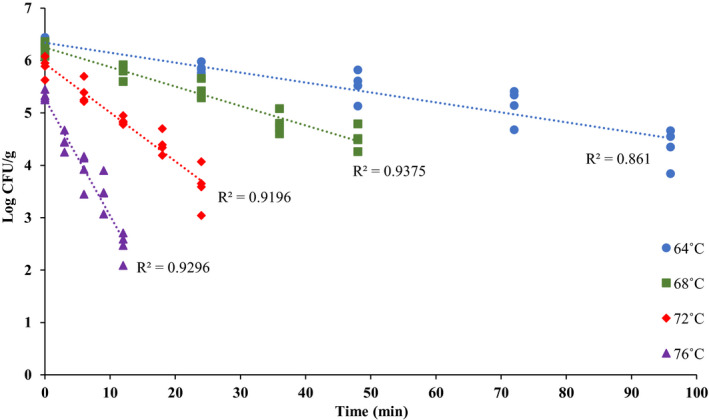
*Salmonella* population [Log CFU/g; mean and three replications] versus time (min) graphs used for calculating *D*‐values at 64, 68, 72 and 76°C

**FIGURE 9:**
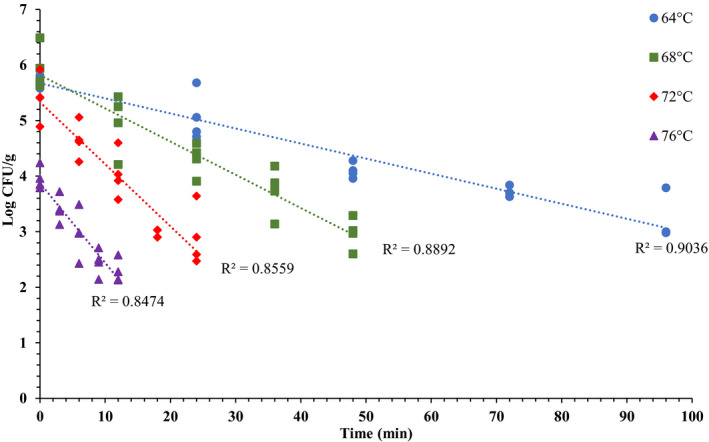
*L. monocytogenes* population [Log CFU/g; mean and three replications] versus time (min) graphs used for calculating *D*‐values at 64, 68, 72, and 76°C

**FIGURE 10:**
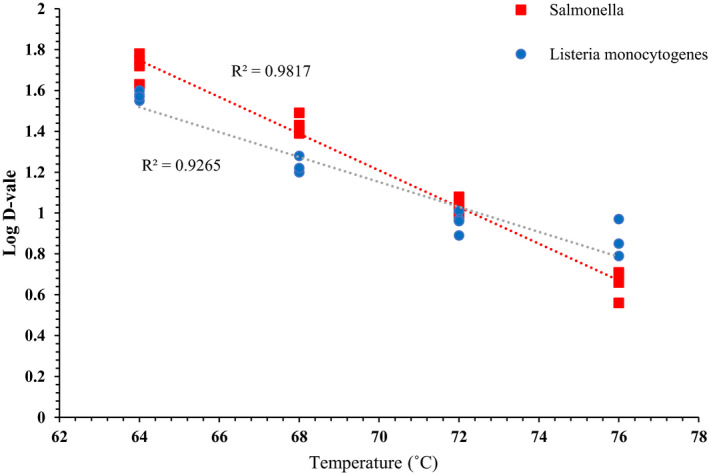
Log *D*‐values [mean and three replications] versus temperature (°C) graphs used for calculating *z*‐values for S*almonella* and *L. monocytogenes*

Thermal inactivation graphs used to calculate the *D*‐values of *Salmonella* and *L. monocytogenes* in brownie batter at 64, 68, 72, and 76°C are presented in Figures 8 and 9. The log *D*‐values versus temperature graphs to calculate *z*‐values are presented in Figure 10. These temperatures were selected for the *D*‐values determination because at temperatures >76°C, microbial populations decreased to <3.5 log CFU/g by the time target temperatures were achieved (0 min), which was not adequate to plot thermal inactivation graphs. Moreover, microbial kill at temperatures >76°C was very quick resulting in very short sampling time intervals. Whereas, at temperatures <64°C, microbial kill was very slow resulting in very long thermal treatments. The calculated mean *D*‐ and *z*‐values in brownie batter are presented in Table 3. Overall, *Salmonella* *D*‐values were greater at 64, 68, and 72°C compared to that of *L. monocytogenes*; but at 76°C, *L. monocytogenes* *D*‐values were greater. The *z*‐values for *Salmonella* and *Listeria monocytogenes* were 16.4 and 11.1°C, respectively.

**TABLE 3:** Mean (± SE; *n* = 3) *D*‐values (min) and *z*‐values (°C) of *Salmonella* and *L. monocytogenes* brownie batter

Item	*Salmonella*	*L. monocytogenes*
64.0°C	53.4 ± 5.38	37.5 ± 1.13
68.0°C	27.2 ± 2.04	16.9 ± 1.12
72.0°C	10.7 ± 0.72	9.1 ± 0.69
76.0°C	4.6 ± 0.49	7.3 ± 1.08
*z*‐Value	11.1 ± 0.71	16.4 ± 0.90

In a similar study, Channaiah et al. (2016) reported that the *D*‐values of 3‐serovar *Salmonella* cocktail (Typhimurium, Newport and Senftenberg 775W) in hamburger bun dough at 55, 58, and 61°C were 28.64, 7.61 and 3.14 min, respectively, and the *z*‐value was 6.58°C. Whereas, Channaiah et al. (2017) reported that the *D*‐values of 3‐serovar *Salmonella* cocktail in muffin batter at 55, 58, and 61°C were 62.2, 40.1, and 16.5 min, respectively, and the *z*‐value was 10.4°C. In a different study, Channaiah et al. (2018) determined the *D*‐values of 7‐serovar *Salmonella* cocktail (Newport, Typhimurium, Senftenberg, Tennessee and three dry food isolates) in donut dough. They reported that the *D*‐values of *Salmonella* cocktail were 8.6, 2.9, and 2.1 min at 55, 58, and 61°C, respectively, with the *z*‐value of 10°C. The differences in the *D*‐values of *Salmonella* in the current study and that of Channaiah et al. (2016, 2017, 2018) can be attributed to the different *Salmonella* serovars used and the differences in the chemical/nutrient composition of the products used in these studies. In general, with the increase in fat, protein and sugar content of products, the thermal resistance of microorganisms also increases. Fat, protein, and sugar molecules have the capability to form a protective coating around the bacterial cell (Juneja et al., 2001; Mattick et al., 2001; Smith et al., 2001). This protective coating acts as a sheath against the heat effect and subsequently leading to elevated thermal resistance values. For example, Sekhon et al. (2020) determined the thermal resistance of *Salmonella* cocktail (similar to this study) in nonfat dry milk and whole milk powder during the storage period of 6 months. Sekhon et al. (2020) reported that although the thermal resistance of *Salmonella* cocktail in whole milk powder (higher fat content) and nonfat dry milk was similar at the start of the storage, the thermal resistance of *Salmonella* increased during the storage period in whole milk powder but stayed similar in nonfat dry milk.

## 4 CONCLUSIONS

The current research showed that a typical commercial brownie baking process with >100°C internal temperature for at least 40 min will result in ≥5‐log reductions in *Salmonella* and *L. monocytogenes*. It should be noted that validation studies are specific to the individual pathogens in specific food formulation and process. Therefore, independent validation studies should be conducted for other pathogens or if significant changes are made to the brownie recipe used in the current study. The thermal resistance of bacteria immensely depends on the pH and a_w_ of the food matrix; therefore, the continuous determination of pH and a_w_ values during the baking process is critical in studying the survivability of respective pathogens. The *D*‐values calculated in the current study can be used as the bases to develop the thermal inactivation predictive models for the inactivation of *L. monocytogenes* and *Salmonella* in brownies. However, additional *D*‐ and *z*‐value data should be calculated to develop strong and robust inactivation models.

## CONFLICT OF INTEREST

The authors do not have any conflict of interest.

## ETHICAL APPROVAL

This study does not involve any human or animal testing.
